# Editorial: The role of TGF-beta superfamily members in immune homeostasis and disease

**DOI:** 10.3389/fimmu.2025.1663433

**Published:** 2025-07-24

**Authors:** Natalie Eva Nieuwenhuizen, Ioannis Eleftherianos, Piotr Jan Kraj, Maria Semitekolou

**Affiliations:** ^1^ Institute of Hygiene and Microbiology, Julius Maximilian University of Würzburg, Würzburg, Germany; ^2^ Infection and Innate Immunity Laboratory, Department of Biological Sciences, The George Washington University, Washington, DC, United States; ^3^ Department of Biological Sciences, Old Dominion University, Norfolk, VA, United States; ^4^ Center for Basic Research, Biomedical Research Foundation of the Academy of Athens, Athens, Greece

**Keywords:** TGF-beta superfamily, activin A, bone morphogenetic proteins (BMPs), growth/differentiation factors (GDFs), cancer, inflammation, T cell, Treg

The Transforming Growth Factor beta (TGF-beta) superfamily, encompassing molecules such as TGFs, activins, Bone Morphogenetic Proteins (BMPs), Growth/Differentiation Factor (GDFs) and Nodals, represents the largest family of growth and differentiation factors, playing crucial roles in developmental and physiological processes across animal species (1). These molecules are integral to tissue homeostasis and cell fate determination. Among them, TGF-beta is particularly noted for its regulatory influence on immune responses and tissue fibrosis (2, 3). Recent research has expanded our understanding of the immune functions of other superfamily members, including activin A and BMPs (4). These molecules signal through receptor complexes composed of type I and II serine/threonine kinase receptors, which activate Smads and initiate transcription. The functional diversity of TGF-beta superfamily ligands is attributed to the varied combinations of receptor complexes and the interaction of Smads with numerous transcription factors and co-regulators (5). Despite growing evidence of their roles in immune responses, the involvement of these cytokines in diseases, especially infectious diseases, remains underexplored. This gap is partly due to the embryonic lethality of gene deletions and the challenges in detecting their roles through transcriptomic approaches (6). However, advancements in genetic technologies, such as CRISPR/Cas9 and specific inhibitors, offer promising avenues to uncover novel functions of these molecules (7).

This Research Topic aimed to elucidate the roles of TGF-beta superfamily members in immune homeostasis and disease. The primary objectives included investigating the signaling pathways of these molecules, understanding their modulation of immune responses, and exploring their involvement in various diseases. Specific questions that were addressed included the mechanisms by which these cytokines influence immune responses and their potential roles in infectious diseases, autoimmunity, allergy, inflammation, and cancer as well as the potential therapeutic targeting of TGF-beta superfamily molecules.

The role of Activin A in neutrophil function is not well understood. Divolis et al. generated a novel neutrophil-specific Activin A-deficient mouse model to study its role in the immune response to Influenza A virus (IAV) infection. They showed that Activin A deficiency in neutrophils leads to severe inflammation and hemorrhagic histopathology in the lungs of IAV-infected mice. This phenotype was further linked to excessive production of neutrophil extracellular traps (NETs). These results indicate that Activin A produced by activated neutrophils regulates their pro-NETotic predisposition, a process which contributes to the reduction of accompanying tissue damage that is induced by neutrophil overactivation.

Along similar lines, Dai et al. examined the role of Growth differentiation factor 15 (GDF15) in macrophage-mediated inflammatory responses. In a previous study, the authors detected a subset of macrophage cells in human lung tissues that expressed GDF15 at high levels (GDF15^high^ macrophages). Here, the researchers examined whether the GDF15^high^ macrophages represent a functionally distinct population with anti-inflammatory properties. First, they showed that macrophages derived from human peripheral blood mononuclear cells contain a small number of GDF15^high^ macrophages that represent a distinct population, and then using single-cell RNA sequencing (scRNA-seq) they characterized these cells molecularly and determined the anti-inflammatory function of the *in vitro* derived GDF15^high^ cells.

Two articles in the Research Topic describe the role of TGF-beta family members in anti-tumor immunity. The first work, by O’Connor et al., examines whether cancer-associated fibroblasts (CAF) directly induce T cell production of the chemokine CXCL13, which is generally associated with beneficial anti-tumor responses and the formation of tertiary lymphoid tissue. One of the key findings of this work is that suppression of TGF-β signaling prevents the CAF-driven upregulation of CXCL13 as well as the expansion of regulatory T cells. This work concludes that these processes may affect sensitivity to immunotherapy and their intricacies could be explored for the development of novel therapeutics against cancer. The second work by Pinjusic et al. used single-cell RNA sequencing to examine the effect of Activin A in the syngeneic mouse melanoma model YUMM3.3. The researchers show that Activin A remodeled the immune cell landscape and promoted melanoma growth via the STING pathway. This information reveals a novel Activin A-dependent mechanism that regulates anti-tumor function in melanoma.

The Research Topic includes two reviews and a hypothesis article. The first review by Isik et al. focuses on the role of the immunomodulatory factor GDF15 in neurodegeneration. The authors included information suggesting that elevated GDF15 is associated with neurodegenerative disease and injury. They performed a comprehensive literature search to conclude that GDF15 is involved in neuroprotective molecular mechanisms in neurodegeneration. It may promote functional recovery by attenuating neuron loss and damage, and regulates peripheral rather than local neuroinflammatory responses. The second review by Wang et al. covers important information on the potential of TGF-beta inhibitors for prevention and treatment of liver fibrosis. The authors refer to the relationship between the TGF-beta signaling pathway and liver fibrosis, provide a detailed account of the involvement of TGF-beta signaling in liver fibrosis, and outline recent progress on the design and use of various types of TGF-beta inhibitors in liver fibrosis and liver cancer treatment. Finally, the hypothesis article by Elkoshi lays out the evidence for the participation of TGF-β in metabolic dysregulation and further explores a connection between the TGF-β/Smad pathway, IL-1β and IL-6 levels, metabolic pathways, allergic disease subtypes, inflammation and the risk of cancer within a unified framework, highlighting a potential role for Th17.

The published review and research articles in this Research Topic present important findings and insights into the complex roles of TGF-beta superfamily members in immunity and disease. This information contributes toward a better understanding of TGF-beta signaling regulation, the modulation of immune responses by TGF-beta superfamily members and their roles in infectious disease, autoimmunity, allergy, inflammation, and cancer. This knowledge is critical for dissecting the expression profiles of TGF-beta superfamily components in different diseases and targeting of TGF-beta superfamily molecules for host-directed therapies. We thank all the authors, reviewers and editors for their assistance in successfully completing this Research Topic.
